# Ultrasound-guided totally implantable venous access ports via the right innominate vein: a new approach for patients with breast cancer

**DOI:** 10.1186/s12957-019-1727-0

**Published:** 2019-11-25

**Authors:** Liang Xu, Wenming Qin, Weiwei Zheng, Xingwei Sun

**Affiliations:** 1 Department of General Surgery, Changshu Hospital Affiliated to Nanjing University of Chinese Medicine, Changshu, 215500 Jiangsu China; 2 Department of Pain, Bazhong Central Hospital, Bazhong, 636000 Sichuan China; 3 Department of Orthopaedics, Affiliated Suzhou Hospital of Nanjing Medical University, Suzhou, 215004 Jiangsu China; 4 Department of Intervention, The Second Affiliated Hospital of Soochow University, Suzhou, 215004 Jiangsu China

**Keywords:** Totally implantable venous access ports, Ultrasound-guided, Innominate vein, Complications, Breast cancer

## Abstract

**Background:**

To evaluate the feasibility and safety of ultrasound-guided totally implantable venous access port (TIVAP) implantation via the right innominate vein in patients with breast cancer.

**Methods:**

Sixty-seven breast cancer patients underwent ultrasound-guided implantation of TIVAPs via the right innominate vein for administration of chemotherapy. Clinical data including technical success, success rate for the first attempt, periprocedural, and postoperative complications were recorded and retrospectively studied.

**Results:**

All patients underwent successful surgery. The success rate of the first attempt was 95.52% (64/67). The operation time was 28 to 45 min, with an average of 36 ± 6 min. Periprocedural complications included artery punctures in 1 (1.50%, 1/67) patient. Prior to this study, the mean TIVAP time was 257 ± 3 days (range 41 to 705 days). The rate of postoperative complications was 4.48% (3/67), including catheter-related infections in 1 case and fibrin sheath formation in 2 cases. Up to the present study, three people had unplanned port withdrawal due to complications, and the TIVAPs for 25 patients were still in normal use.

**Conclusions:**

The success rate of ultrasound-guided TIVAPs via the right innominate vein is high with low complications, thus safe and feasible. This technique can provide a new option for chemotherapy of breast cancer patients.

## Background

Totally implantable venous access ports (TIVAPs) can be used for the infusion of various chemotherapeutic drugs [1]. Compared with peripherally inserted central catheters (PICCs), TIVAPs are widely used in the clinic because of their advantages of more convenient care and lower rate of complications [2]. Currently, the application of TIVAPs through either the internal jugular vein (IJV) or subclavian vein (SCV) or cephalic vein surgical cutdown approach is the most widely used [3, 4].

Recently, ultrasound-guided central vena catheterization (CVC) via the innominate vein (INV) has been suggested as an alternative approach to IJV. It is safe and reliable both in infants and adults [5–9]. However, the INV approach is rarely reported for TIVAPs, specifically in breast cancer patients, and it is still overlooked [10].

To evaluate the feasibility and safety of this new approach for TIVAPs, clinical data of patients receiving TIVAPs via the right INV for chemotherapy were collected and retrospectively analyzed.

## Methods

### Patients

Clinical and nursing data for 67 adult patients with left breast cancer who underwent implantations of TIVAPs via the right INV from January 2017 to January 2018 were collected (Table 1). The TIVAP used was purchased from B. Braun (B. Braun, 04436946 6.5F, FR).

**Table 1:** Patients’ characteristics (*N* = 67)

Characteristics	*N* (%)
Age (years) (mean ± SD)	46 ± 11 (35–63)
Female/male	67/0
Body weight (kg) (mean ± SD)	52 ± 16 (43–79)
Height (cm) (mean ± SD)	163 ± 19 (150–171)
Body mass index (mean ± SD)	21 ± 4 (19–25)

### Implantation procedures

The study was approved by the ethics committee of the Changshu Hospital Affiliated to Nanjing University of Chinese Medicine, and all methods were performed in accordance with the relevant guidelines and regulations.

Preoperative blood routine examination and blood coagulation tests were performed. Surgery was performed in strict accordance with aseptic operation procedures, such as hand washing and disinfection during the operation.

Patients lay supine with heads turned to the left, and the surgeon stood at the right side of the patient’s head with the high-frequency ultrasound probe in his left hand and the puncture needle in his right.

**Fig. 1:**
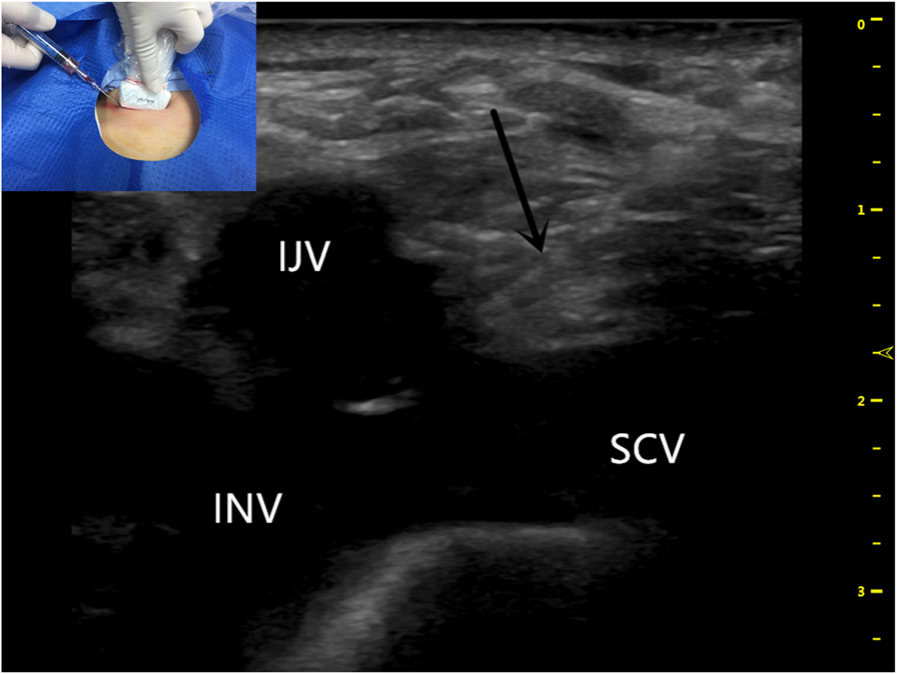
Ultrasound-guided successful puncture of right INV with an inserted needle (black arrow). INV longitudinal view, in-plane approach. INV indicates innominate vein. IJV indicates internal jugular vein. SCV indicates subclavian vein

The ultrasound probe went down the right IJV to the superior sternoclavicular joint, showing that the right IJV and right SCV converged to form the initial part of the right INV, and there was a good view of the right subclavian artery (SCA) (beside the SCV). Then, the right INV was punctured under ultrasound guidance by in-plane technique (Fig. 1).

After the successful puncture, the introducer sheath and port catheter were introduced subsequently. A subcutaneous pocket was prepared by blunt dissection on the anterior chest wall, and the pocket was sized to just fit the port. A tunnel needle catheter traction crossed above the clavicle through the incision, and the catheter was cut in a suitable position with fluoroscopy and joined to the port.

**Fig. 2:**
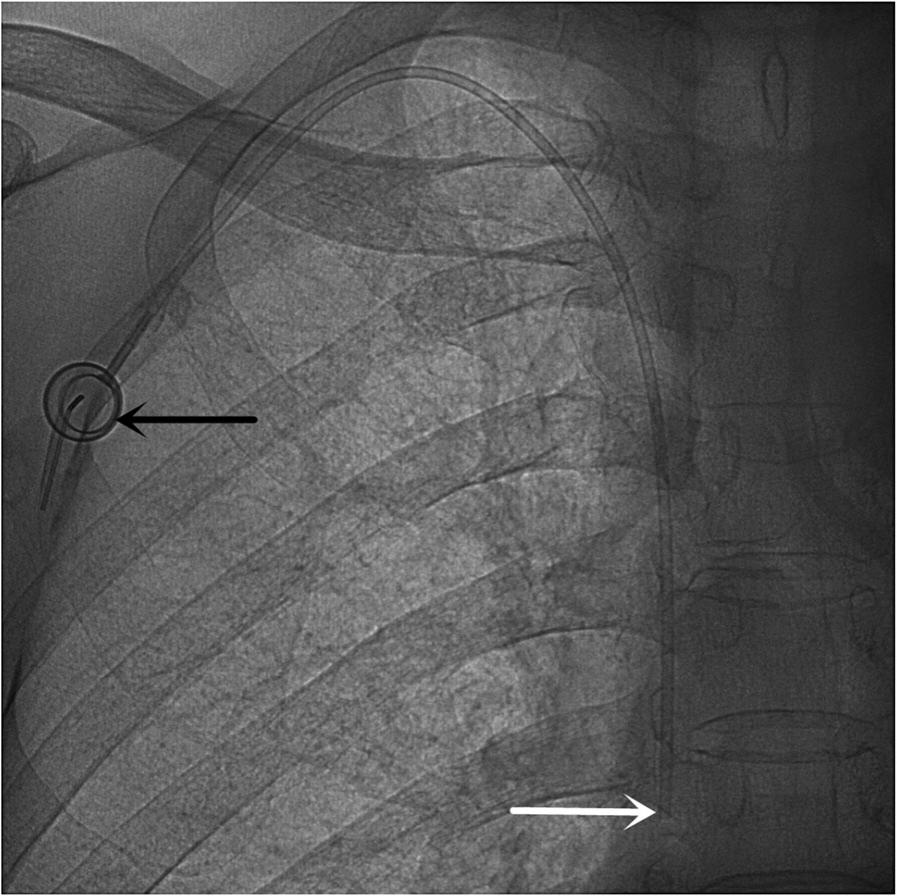
TIVAP is implanted via the right INV approach, crossing over the right clavicle. The port (black arrow) is on the right chest wall, and the catheter tip (white arrow) is placed at the joint of the superior vena cava and the right atrium

Subsequently, the incision was sewn up and covered with a compression bandage. The infusion was verified by puncturing the port and drawing back. The correct position of the port catheter was assessed again (Fig. 2).

Clinical data including success rate for the first puncture, operation time, perioperative complications, and postoperative complications were recorded.

Specialized nurses were equipped to maintain and manage the TIVAPs after implantation, and a 10-ml flushing tube of 50-100 iu/ml heparin saline or saline was used, not more than once every 28 days.

## Results

The operation was successful in 67 cases (Table 2). The intraoperative puncture needle entered the right INV once successfully to consider the puncture successful at the first puncture. The success rate for the first puncture was 95.52% (64/67). The other three patients had a successful puncture at the second attempt.

**Table 2:** Details of the US-guided right INV puncture for TIVADs (*N* = 67)

Details	*N* (%)
Success rate of surgery (%)	67 (100)
Success rate of first attempt (%)	64 (95.52)
Operation time (minutes) (mean ± SD)	36 ± 6 (28–45)
Length of catheter introduction (cm) (mean ± SD)	19 ± 3 (17–24)
TIVAP time (days)	257 ± 39 (41–605)

The operation time was 28 to 45 min, with an average of 36 ± 6 min. The length of the catheter was 17 to 24 cm, with an average of 19 ± 3 cm. Prior to this study, the mean TIVAP time was 257 ± 39 days (range 41 to 605 days) (Table 2**)**.

Perioperative complications included one case (1.50%, 1/67), in whom the SCA was mistakenly perforated, but the second puncture was successful with no interventions. No pneumothorax or hemothorax were observed **(**Table 3**)**. Postoperative complications included mean complications that occurred 2 weeks after implantation. The postoperative complications rate was 4.5% (3/67), including catheter-related infection in 1 case and fibrin sheath formation in 2 cases. They all led to unplanned port withdrawal after failure of anti-infection or thrombolytic therapy **(**Table 3**)**.

**Table 3:** Incidence of intraoperative and postoperative complications and actions taken (*N* = 67)

Complications	No. (%)	Actions taken
Artery perforated	1 (1.5)	Self-limited
Catheter-related infection	1 (1.5)	Antibiotics and port removal
Fibrin formation	2 (3.0)	Thrombolysis and port removal
Total	4 (6.0)	

Notably, no catheter ectopic or catheter rupture occurred during the follow-up period. Twenty-five TIVAPs were still in normal use at the end of the follow-up period.

## Discussion

TIVAPs are widely used in the clinic because they require no external dressing, allow patient activity, and improve the life of patients, especially in patients with breast cancer who require frequent chemotherapy and blood sampling [11]. At present, surgical cutdown of the cephalic vein and percutaneous puncture for TIVAP implantation via IJV and SCV are the most widely used for their high success rate and low complications [3, 12, 13].

However, the IJV and SCV may not be the best option for many clinical situations. The high puncture point seems to be one of the shortcomings of the IJV approach, the large angle of the fold of the catheter due to the high puncture point may lead to catheter discount, clogging, and fracture [14]. Larger angle and longer catheterization pathway are important factors in the reduction of patient comfort and may cause an unattractive appearance for patients with breast cancer after TIVAP placement [15]. The puncture point of SCV approach is lower, which is more convenient and comfortable than IJV, but the occurrence of pinch-off syndrome (POS) may lead to dysfunction of the catheter. POS is the main cause of catheter rupture or fracture for the SCV approach [14].

In 1982, Niederhuber et al. of MD Anderson Cancer Center in the USA, first applied the TIVAP surgical technique to the central vein through the cephalic vein [16]. Studies showed that, compared with the SCV approach, the incidence of complications of the INV approach using surgical techniques for TIVAP is low, and it is considered superior to the SCV approach [17]. Koketsu et al. [18, 19] also believed that TIVAP could provide safe and feasible infusion channels for patients through INV, which is worthy of promotion and application. However, surgical incision and implantation of TIVAPs also have the disadvantages of long operation time, low success rate, and great trauma [20].

With the development of ultrasound technology, however, ultrasound-guided INV catheterization has been gradually applied in clinical practice, and many studies have confirmed its safety and effectiveness [5–9]. However, ultrasound-guided puncture of INV for TIVAPs is rarely reported and is still overlooked.

The IJV merges with the SCV behind the sternoclavicular joint to form the INV, and the bilateral INVs converge to form the superior vena cava (SVC). We know that the INV is relatively fixed and has a larger diameter than the IJV and SCV; this provides the possibility of ultrasound-guided puncture of INV safely and effectively [21].

In this preliminary study, the right INV approach was obtained only, as the thoracic duct afflux into the central vein is via left INV, so the right INV approach was preferred to avoid thoracic duct damage. But in Beccaria’s study [9], 78 patients with left INV catheterization did not have thoracic duct injury. Another study showed that left INV catheterization was safe and feasible in children [22]. The safety and feasibility of left INV approach of TIVAPs for patients need to be studied further.

Beccaria et al. conducted a comparative study between CVC via the INV and IJV approach. The study indicates that the US-guided CVC via INV approach is safe and easy to operate, and it is a reasonable alternative to IJV approach in adults [9]. In our study, right INV approach was adopted in 67 cases, the success rate of the first puncture was 95.52% (64/67), similar to the results of 90.18% (257/285) reported by Beccaria et al [9]

One study showed that the success rate of catheterization of the left INV was higher than that of the right in newborns and children [23].

In this study, the rate of perioperative complications was 1.50% (1/67), consisting of one case of self-limited arterial puncture, visualized as SCV by ultrasound. The second puncture was successful, and there was no hematoma formation.

The overall postoperative complication rate in this study was 4.48% (3/67), which was lower than that in most other studies [12, 24]. Catheter-related infections were found in 1 case 5 weeks after surgery. The blood culture showed *Staphylococcus aureus*. Fibrin sheath formation was found by digital subtraction angiography (DSA) in 2 cases. They all led to unplanned port withdrawal after active anti-infection and failure of thrombolytic therapy. It remained important to avoid unplanned port withdrawal by standardizing the operation and paying attention to the maintenance and management of the catheter.

Lin et al. [25] reported 2620 cases of patients with the SCV puncture route. The incidence of catheter fracture was 2.6%, highlighting that POS is the main cause of catheter rupture. In this study, supraclavicular puncture of right INV was used to avoid the occurrence of POS by crossing above the clavicle, and no catheter fracture was found. In addition, none of the patients had catheter malposition after the procedure, which might be related to the fact that the range of catheter activity by INV approach was small, and the location of the catheter was accurately positioned by DSA fluoroscopy during the operation.

Given the preliminary results reported here (the study was retrospective and the cases were limited), there is a clear need for a randomized controlled study to confirm the feasibility and safety of the ultrasound-guided right INV approach for TIVAPs. It may stimulate future research in this area.

## Conclusions

TIVAPs are widely used in clinical practice to avoid repeated venipuncture and increase the freedom of activity for the patient. Ultrasound-guided supraclavicular TIVAPs through the right INV approach avoid the large angle bending of catheter and POS, have a high success rate, and show low complications, thereby providing another option for patients with breast cancer.

## Data Availability

Research data can be obtained from corresponding author upon reasonable request.

## Abbreviations

CVC: Central vena catheterization
DSA: Digital subtraction angiography
IJV: Internal jugular vein
INV: Innominate vein
PICC: Peripherally inserted central catheters
POS: Pinch-off syndrome
SCA: Subclavian artery
SCV: Subclavian vein
TIVAP: Totally implantable venous access port
